# CT-707 overcomes hypoxia-mediated sorafenib resistance in Hepatocellular carcinoma by inhibiting YAP signaling

**DOI:** 10.1186/s12885-022-09520-5

**Published:** 2022-04-19

**Authors:** Zibo Chen, Tao Yuan, Fangjie Yan, Song Ye, Qin Xie, Bo Zhang, Nengmin Lin, Qiaojun He, Bo Yang, Hong Zhu

**Affiliations:** 1 Zhejiang Province Key Laboratory of Anti-Cancer Drug Research, College of Pharmaceutical Sciences, Zhejiang University, Hangzhou, China; 2 Frederick National Laboratory for Cancer Research, National Cancer Institute, National Institutes of Health, Frederick, USA; 3 Innovation Institute for Artificial Intelligence in Medicine, Zhejiang University, Hangzhou, China; 4 School of Medicine, Second Affiliated Hospital, Zhejiang University, Hangzhou, Zhejiang China; 5 Cancer Center of Zhejiang University, Hangzhou, China; 6 Department of Clinical Pharmacology, Key Laboratory of Clinical Cancer Pharmacology and Toxicology Research of Zhejiang Province, Affiliated Hangzhou First People’s Hospital, Zhejiang University School of Medicine, Hangzhou, China

**Keywords:** CT707, Sorafenib resistance, YAP, Hypoxia, Hepatocellular Carcinoma

## Abstract

**Background:**

Hepatocellular carcinoma (HCC) is one of the leading causes of cancer-related deaths worldwide. Sorafenib is the first-line treatment for advanced HCC, but the anti-cancer effects remain to be improved as indicated by its low response rates and failure to prolong the progression-free survival (PFS). Thus, it is urgent to explore approaches to improve the clinical outcome.

**Materials and methods:**

The effect of Sorafenib in HCC was analyzed by SRB (sulforhodamine B) assay in normoxia and hypoxia, respectively. The different dose combination effect of CT707 and sorafenib was analyzed by SRB assay in hypoxia. Flow cytometry assay was used to detect the cell apoptosis rate with CT707 and sorafenib treatment in hypoxia. Western blotting was used to detect the expression levels of apoptosis -related proteins and the mechanism of CT707 overcome the resistance of sorafenib in hypoxia.

**Results:**

Our study showed that the characteristic intratumor hypoxia of advanced HCC is one of the major factors which mediated the drug resistance towards sorafenib in HCC. And CT-707, a novel multi-kinase inhibitor, could sensitize the hypoxic HCC cells towards sorafenib. Further studies showed that CT-707 abolished the nuclear translocation of Yes Associate-Protein (YAP), which has been demonstrated as one of mechanism of hypoxia-mediated sorafenib-resistance in HCC.

**Conclusions:**

Overall, this study not only favors the development of this novel multi-kinase inhibitor CT-707 as a therapeutic agent against HCC, but also provides a potential strategy to overcome the hypoxia-mediated resistance to sorafenib in HCC patients.

**Supplementary Information:**

The online version contains supplementary material available at 10.1186/s12885-022-09520-5.

## Introduction

Hepatocellular carcinoma (HCC) is the leading cause of cancer-related deaths malignant solid tumor worldwide [1, 2]. Most HCC patients are diagnosed at advanced stage, which are generally suffered from the low efficiency to hepatic resection and liver transplantation therapy [3], therefore largely dependent on the systematic therapy including multi-kinase inhibitors or immunotherapy. Sorafenib, a multikinase inhibitor mainly targeting vascular endothelial growth factor receptors (VEGFR) 1–3, is the firstly-approved first-line kinase inhibitor for advance HCC treatment [4]. Nevertheless, a large number of HCC patients have low response towards sorafenib, or develop resistance to sorafenib very soon, and the progression free survival (PFS) almost remained unchanged with sorafenib treatment [5]. Several lines of evidence have implicated that intratumor hypoxia plays critical roles to the low efficacy of sorafenib in HCC patients [6–8]. Particularly, our previous study reveals that hypoxia-mediates resistance of sorafenib in HCC is conferred by the nuclear translocation of Yes-associated protein (YAP) which promoted cell survival and escaped apoptosis [9].

YAP is a transcriptional co-activator negatively-regulated by the upstream components of Hippo pathway, nuclear translocation of YAP would transactivate a variety of target genes to promote cell proliferation and inhibit apoptosis [10]. The aberrant over-activation of YAP was extensively observed in HCC tumor samples; particularly, in those hypoxic regions [11]. Therefore, the dysregulation of YAP not only leads to tumorigenesis under normoxia, but also plays indispensable roles in hypoxia-mediated malignant progression, including hypoxia-mediated sorafenib resistance [12]. In our recent study, we utilized a cell-based YAP-TEADs luciferase reporter system and identified a multi-kinase inhibitor CT-707, a novel anticancer drug candidate approved by National Medical Products Administration (NMPA) for phase I clinical trial (NCT02695550), as an novel YAP inhibitor possessing enhanced anti-cancer activity against hypoxic HCC [13], but whether CT-707 could abrogated the hypoxia-mediated resistance towards anti-cancer drugs remains unknown.

In this study, we demonstrated that CT-707 greatly improved the anticancer effects of sorafenib under hypoxia, reinforced the apoptotic population in sorafenib groups. These effects were accompanied with the inhibitory effect against YAP activity by CT-707. Given that CT-707 is multi-kinase inhibitor undergoing clinical trial, our findings demonstrate that this agent could overcome the hypoxia-mediated sorafenib resistance, provide a promising therapeutic strategy for the treatment of those HCC patients suffering from sorafenib resistance.

## Materials and Methods

### Materials

Sorafenib was obtained from Aladdin, CT-707 was provided by Centaurus Biopharma. The primary antibodies against cleaved Poly (ADP-ribose) polymerase (PARP), anti-YAP, phospho-YAP (Ser127) were purchased from Cell Signaling Technology. The primary antibody against β-actin and horseradish peroxidase (HRP)-labeled secondary anti-rabbit, anti-goat, and anti-mouse antibodies were purchased from Santa Cruz Biotechnology.

### Cell lines and culture

The human HCC cell lines HepG2, SMMC-7721 and Bel-7402 were purchased from Shanghai Institutes for Biological Sciences (Chinese Academy of Sciences, Shanghai, China). Bel-7402 and SMMC-7721 were normally cultured in RPMI1640 medium (Gibco, Grand Island, NY, USA), all supplement with 10% FBS (Gibco, Grand Island, NY, USA) in a 5% CO_2_ humidified incubator at 37 degrees. And in order to get hypoxic cells. Cells were exposed to hypoxic conditions (1% O_2_) in a hypoxia incubator filled with a mixture of 1% O_2_, 5% CO_2_and 94% N_2_.

### Cell proliferation assay

SMMC-7721 and Bel-7402 cells were treated with various concentrations of CT-707 in the first 24 h and then with various doses of sorafenib for 48 h in normoxic or hypoxic condition, and cell proliferation were measured by microscopy (Leica DM4000 B) and sulforhodamine B (SRB) protein assay (Sigma, S1402) [14]. For SRB, Cells (7000/well) were incubated with 10% trichloroacetic acid (TCA) for 1 h (4 °C) and washed it at least 3 times by water, then stained with sulforhodamine B for 30 min. The sulforhodamine B was washed away with 1% cold acetic acid, and 100 μl of 1% Tris-base was added to each well. The optical density (OD) was determined at 515 nm by a Multiskan Spectrum plate reader (Thermo Electron Corporation, Marietta, OH, USA). For clonogenic assay, Cells were observed and photographed by 10X objective, the results were analyzed by Leica Microscope Imaging Software.

### Flow cytometry

Propidium iodide (PI) staining and annexin V-PI (AV-PI) staining were used to detect the apoptosis of cells by flow cytometry on a FACS Calibur cytometer (Becton Dickinson) as described previously [15].

### Western blot

Cells were lysed with the loading buffer. Proteins were fractionated on 10% SDS-PAGE and transferred to polyvinylidene fluoride membrane (Millipore Corporation). The following antibodies were used: anti-YAP (Cell Signaling Technology, 4912 s), phospho-YAP (Ser127) (Cell Signaling Technology, #4911), β-actin (Santa Cruz, sc-1615), cleaved-PARP (Santa Cruz, sc-7150).

### Statistical analysis

The results are expressed as the mean ± SD of at least 3 independent experiments. Differences between two means were analyzed by student's t-test and were considered statistically significant when *P* < 0.05.

## Results

### Hypoxia mediated sorafenib resistance of HCC cells

**Fig. 1:**
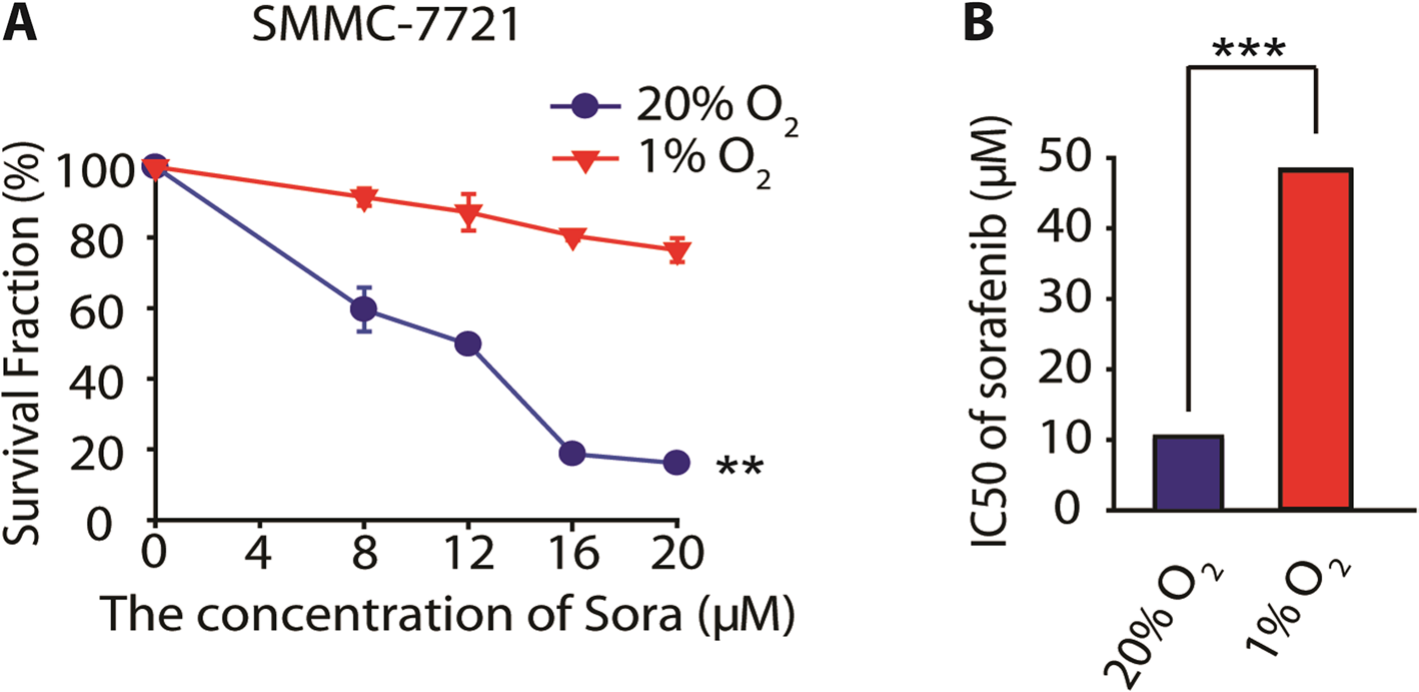
Hypoxia mediated sorafenib resistance in hepatocellular carcinoma. **A** SMMC-7721 cell was treated with serial concentrations of sorafenib in normoxia and hypoxia and cell survival were detected using SRB assay. **B** The IC_50_ values of sorafenib in normoxia and hypoxia. Data are representative of 3 independent experiments and are expressed as the mean ± SD. The symbols refer to ** *P* < 0.01 and *** *P* < 0.001, respectively

First, we confirmed the antitumor effect of the sorafenib in human HCC cell lines, SMMC-7721 and Bel-7402 in normoxia and hypoxia. Cells were treated with serial concentrations of sorafenib for 72 h, and then SRB staining assay was used to detect the survival fractions. The survival curve of sorafenib is shown in Fig. 1A. When cells were exposed to serial concentrations of sorafenib in normoxia or hypoxia, the survival rate of sorafenib in normoxia or hypoxia were determined respectively, and the results suggested that hypoxia significantly reduced the anti-cancer effect of sorafenib. To specifically evaluate the differential antitumor effect in normoxia and hypoxia, we calculated the half maximal inhibitory concentration (IC_50_) [16, 17]. The IC_50_ values of sorafenib in normoxia and hypoxia were 10.01 μM and 47.99 μM respectively (Fig. 1B). Similar results were obtained on Bel-7402 (Supplemental Fig. 1). These data demonstrate that hypoxic microenvironment conferred the resistance of HCC cells towards sorafenib.

### Cytotoxicity of the combination of sorafenib and CT-707 in human HCC cell lines

**Fig. 2:**
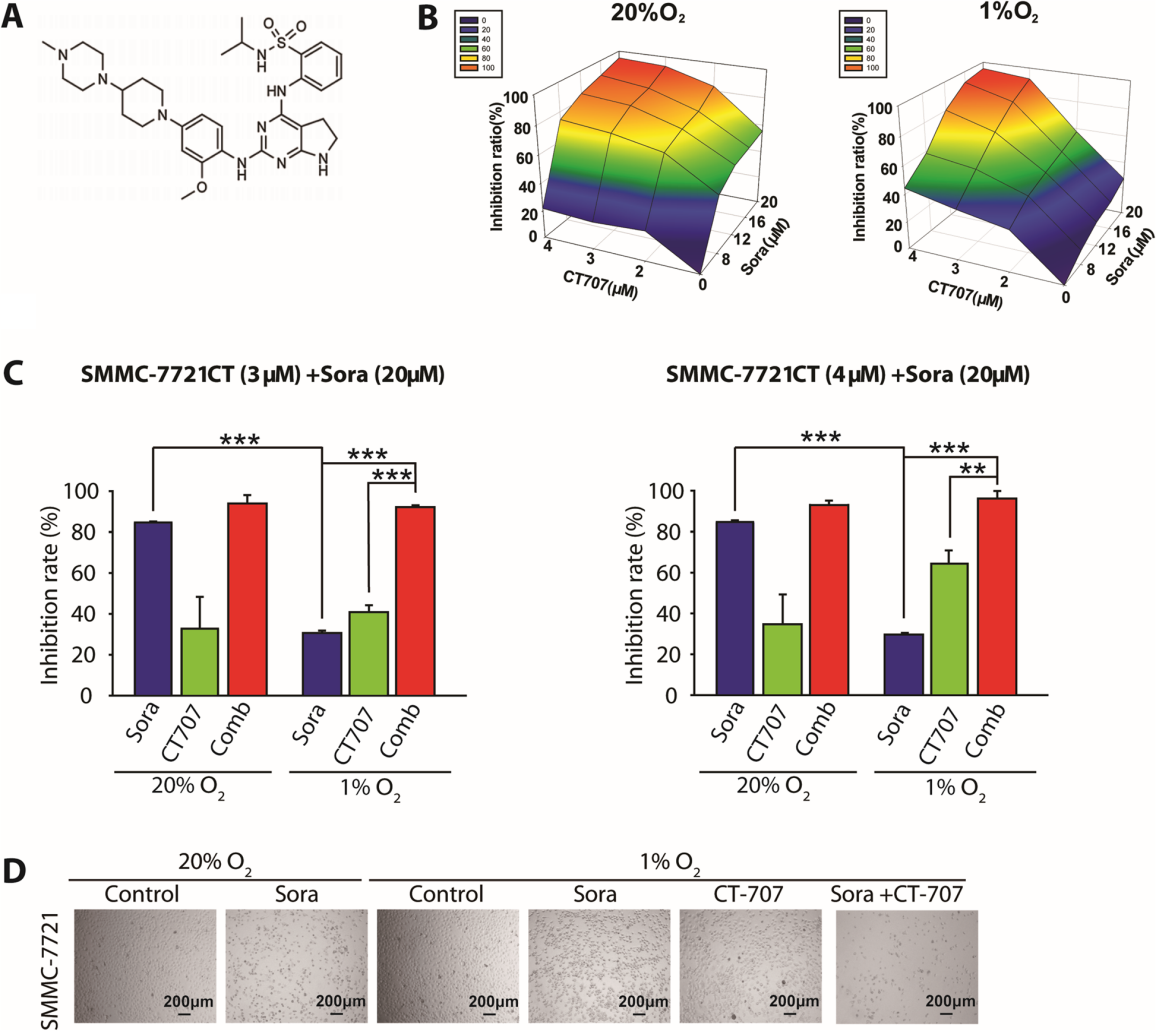
Hypoxia-induced sorafenib resistance can be overcome by CT-707 in vitro. **A** Chemical structures of CT-707. **B**, **C** SMMC-7721 cell was treated with sorafenib at serial concentrations and CT-707 at different concentrations (2 μM, 3 μM, 4 μM), and the inhibition ratio was then determined by SRB assays. Data are representative of 3 independent experiments and are expressed as the mean ± SD. The symbols refer to ** *P* < 0.01 and *** *P* < 0.001, respectively. **D** SMMC-7721 cell was treated with sorafenib (10 μM), CT-707(3 μM) or both, and the cell density was observed by optical microscope

Our previous study has uncovered the enhanced anti-cancer capacity of CT-707 (Fig. 2A) against the hypoxic HCC cells [13]; therefore, we were encouraged to ask whether the combination of sorafenib and CT-707 would exert enhanced effects under hypoxia. Normoxic or hypoxic SMMC-7721 cells were pre-treated with serial concentrations of CT-707 for the first 24 h, and then subjected to serial concentrations of sorafenib for the next 48 h. SRB assay was used to monitor the survival fractions of each group. As shown in Fig. 2B, the combination of sorafenib and CT-707 achieved synergistic anti-cancer effects under hypoxia, which was greater than that under normoxia. Specifically, when cells were exposed to sorafenib (20 μM), CT-707 (3 μM), or their combination in normoxia, the inhibition rates were 83.94%, 32.07%, and 92.94%, respectively. In the contrast, those under hypoxia were 29.76%, 40.32%, and 91.29%, respectively (Fig. 2C). These data revealed that the hypoxia-mediated resistance was remarkably attenuated by CT-707, and the combination of these two agents elicited robustly enhanced anti-cancer activities against hypoxic HCC cells than that under normoxia. Similar results were also achieved when cells were co-exposed to CT-707 at 4 μM (Fig. 2C).

To further demonstrate that CT-707 could overcome the hypoxia-mediated resistance of sorafenib, we treated SMMC-7721 cells with sorafenib (10 μM), CT-707 (3 μM) or both under normoxia and hypoxia, respectively; and observed the cell morphology using optical microscope. Representative pictures of SMMC-7721 and Bel-7402 after 72 h in cell culture dishes were displayed in Fig. 2D and Supplemental Fig. 2. Combination treatment resulted in significant inhibition to the proliferation of SMMC-7721 and Bel-7402 under hypoxia, while the mono-treatment induced moderate inhibition. Taken together, these data suggested that the hypoxic resistance of sorafenib in HCC cells could be greatly abolished by CT-707.

### CT-707 treatment strengthens the apoptosis-induction by sorafenib in hypoxic HCC cells

**Fig. 3:**
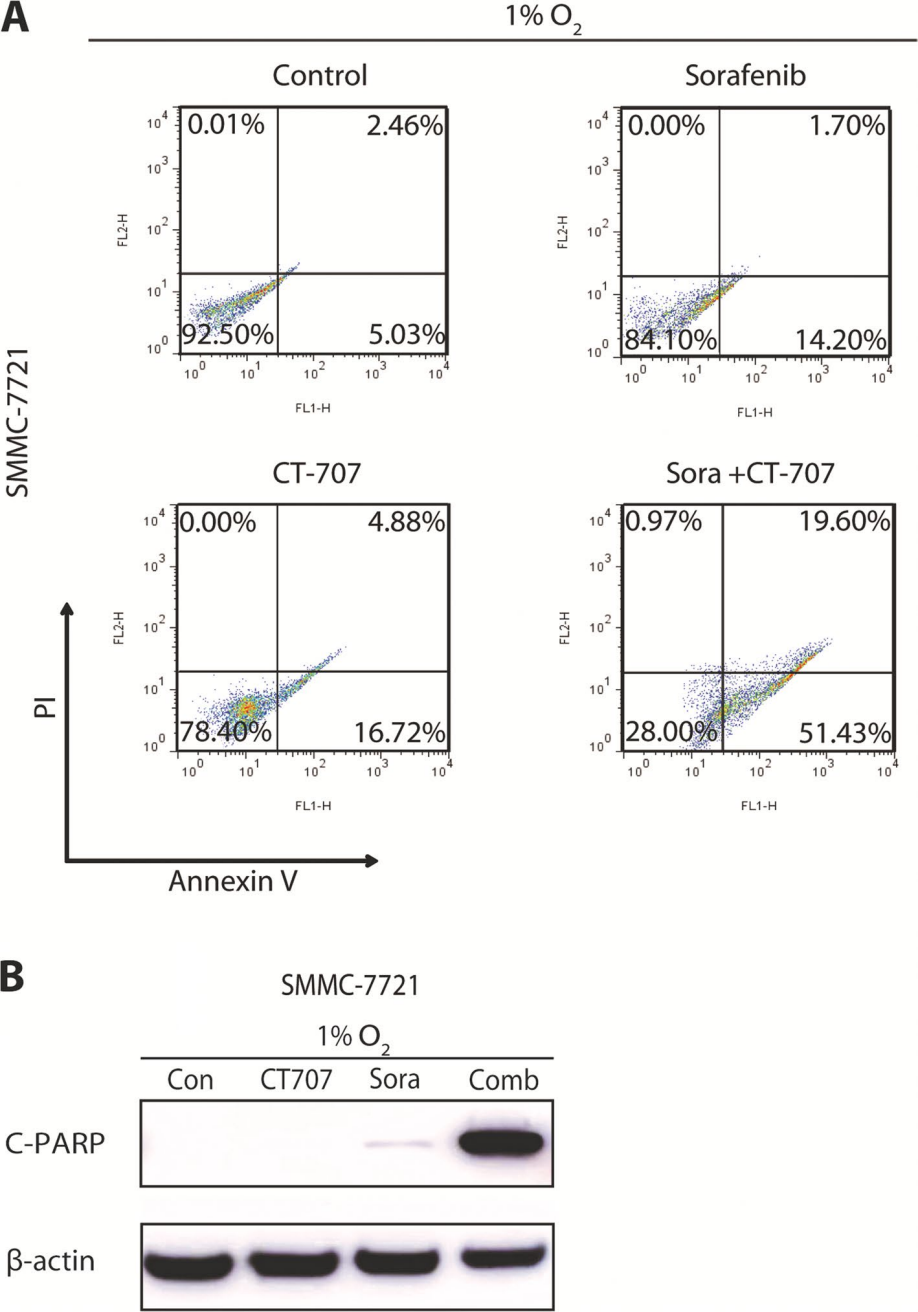
The combination of Sorafenib and CT-707 induced enhanced apoptosis of hepatocellular carcinoma cells. **A** SMMC-7721 cell was treated with sorafenib (15 μM), CT-707(4 μM) or both, and the cell apoptosis was detected by AV/PI staining (the units of the y-axis and x-axis are fluorescence intensity. y-axis: PI staining; x-axis: Annexin V). **B** SMMC-7721 was treated with sorafenib (20 μM), CT-707(3 μM) or both, and the protein expression levels of cleaved PARP (c-PARP) and β-actin in the cell lines were determined using western blot analysis. Full-length blots/gels are presented in Supplementary materials Figure 1A

To further confirm that CT-707 could overcome sorafenib resistance under hypoxia, we assessed the apoptosis of SMMC-7721 and Bel-7402 cells after 72 h treatment by sorafenib (15 μM), CT-707(4 μM) or both. The results detected by PI staining following FACS analysis were shown in Fig. 3A and Supplemental Fig. 3, the apoptosis ratio (early + late apoptosis) of control, sorafenib, CT-707 and combination groups in SMMC-7721 were 7.49%, 15.90%, 21.60% and 71.03% respectively; and those in Bel-7402 were 0.08%, 34.40%, 27.71% and 61.76% respectively. The results indicated that the combination of these two agents enhanced the apoptosis in HCC compared with mono-treatment.

Because most apoptotic cell death undergoes the caspase-dependent pathway [18], we further examine the activation of the caspase cascade of SMMC-7721 cell line after 72-h treatment by sorafenib (20 μM), CT-707(3 μM) or the combination using Western blotting. As demonstrated by Fig. 3A, the combined treatment of CT-707 an sorafenib significantly triggered caspase activation as indicated by the robust cleavage of PARP, the substrate of caspases cascade, which denoting more apoptosis in the combination groups. These findings collectively verified that the combination of CT-707 with sorafenib could significantly enhance the hypoxic anti-cancer activities in HCC.

### CT-707 inhibits YAP nuclear translocation in HCC cell lines

Above-mentioned data illustrated the capability of CT-707 that increased the hypoxic HCC cell susceptibility towards sorafenib. Mounting evidence has implicated the critical roles of YAP signaling in hypoxia-mediated drug resistance [19, 20]. Particularly, our previous studies demonstrated that hypoxia-activated YAP pathway contributed to the decreased drug response of HCC cells towards sorafenib or SN-38 [9, 12]. Based on these findings, we performed a functional screening and identified CT-707 as a novel YAP inhibitor, and this agent possessed superior activity under hypoxia by suppressing hypoxia-induced YAP translocation. Therefore, we next investigated whether CT-707 overcome the resistance to sorafenib through its YAP-inhibitory effect.

**Fig. 4:**
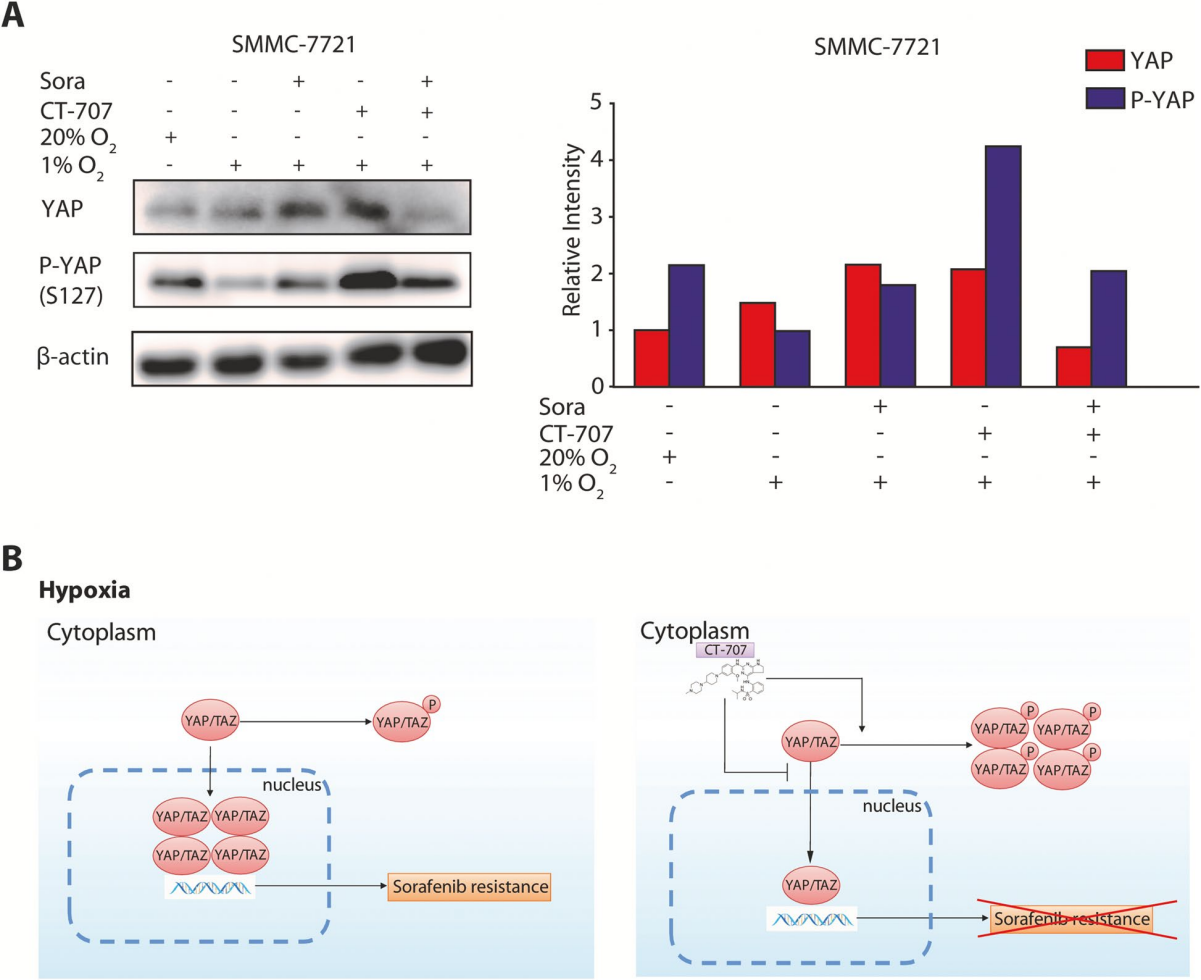
CT-707 increase sorafenib-induced apoptosis under hypoxia by suppressing YAP target genes. **A** The protein levels of YAP, P-YAP and β-actin in SMMC-7721 cell were detected by western blot analysis in the same conditions as Fig. 2 (D). **B** A proposed model of crosstalk among Hippo signaling, sorafenib and CT-707 response in hypoxia. On the left, hypoxia caused decreased levels of p-YAP (Ser127), denoting the nuclear translocation of YAP under hypoxic microenvironment, which induce sorafenib resistance. On the right, CT-707 overcome the resistance of sorafenib in hypoxia by preventing YAP nuclear translocation. Full-length blots/gels are presented in Supplementary materials Figure 1B-D

The core components of the Hippo pathway include the mammalian sterile 20-like kinases (MSTs) and large tumor suppressor kinases (LATSs), impose negative regulation on YAP by phosphorylation on residue Ser127, leading to the cytoplasmic retention of YAP protein. On the contrary, the unphosphorylated YAP would translocate into the nucleus and exert its transactivation function. We assessed the phosphorylated and total levels of YAP in SMMC-7721 cells after 24-h exposure to sorafenib (10 μM), CT-707 (3 μM) or both using Western blot analysis. The results showed that hypoxia caused decreased levels of p-YAP (Ser127), denoting the nuclear translocation of YAP under hypoxic microenvironment (Fig. 4A). While CT-707 exposure under hypoxia significantly induced the phosphorylated YAP, which indicated that in these cells, YAP protein was detained in the cytoplasm (Fig. 4A). These results implied that CT-707 overcome the resistance of sorafenib in hypoxia by preventing YAP nuclear translocation.

## Discussion

HCC is one of the most common cancer in the world. And the incidence of HCC has increased rapidly worldwide in the last decade [21]. The molecular pathogenesis of HCC varies according to the differential genotoxic insults and aetiologias including hepatitis B, C and other confounding factors such as tobacco use, obesity and alcohol abuse [22–24]. Sorafenib is the firstly-approved multikinase inhibitor to treat advanced HCC [25]. Clinical studies have shown some survival benefits on the time to progression (TTP) and overall survival (OS), but the benefits of sorafenib generally could not be sustained for long time treatment due to the acquisition of resistance [26, 27]. Therefore, it is urgent to explore potential strategies to alleviate sorafenib resistance in HCC.

HCC is a type of hypervascular tumor and angiogenesis plays an important role in the development of the disease [28]. Given the sophistication and intricacy of angiogenetic process, it would be extremely difficult for sorafenib to completely suppress blood vessel formation, particularly those microvessels inside HCC tumors [29]. Recent studies showed that Axitinib, a multiple tyrosine kinase inhibitor targeting VEGFR1, VEGFR2, VEGFR3, PDGFR and c-Kit, could improve the anticancer effects of sorafenib in advanced HCC patients [30]. Similar observation was obtained on apatinib, which is also a VEGFR2 inhibitor and showing synergistic effects with sorafenib [31, 32]. Notably, both combination regimens have been evaluated in clinical trials (NCT00678392, NCT02329860).

On the other hand, the clinical outcome of anti-angiogenesis strategies remains debated, recent findings reveal that anti-angiogenic treatment has limited efficacy due to therapy-induced blood vessel alterations, often followed by severe intratumor hypoxia, tumor adaptation, progression and metastasis [33]. Particularly, mounting evidence has established the causal link between hypoxia and reduced susceptibility towards sorafenib. Liang and colleagues found that HIF1α which was induced by hypoxia may contribute to the hypoxic resistance of sorafenib, and EF24, a curcumin analogue, could synergistically strengthen the antitumor effects of sorafenib and overcome sorafenib resistance by inhibiting HIF-1α and promoting proteasomal degradation by up-regulating tumor suppressor Von Hippel-Lindau [8]. Another study conducted by Lin et al*.* uncovers a critical function for METTL3-mediated m^6^A modification in the hypoxic tumor microenvironment and identifies FOXO3 as an important target of m^6^A modification in the resistance of HCC to sorafenib therapy [34]. Our previous study demonstrated that hypoxia induced the nuclear translocation of YAP, subsequently transactivated YAP target genes which promoted cell survival and escaped apoptosis, thereby leading to sorafenib resistance [9, 12].

The important roles of YAP in those HCC tumors presenting hypoxic region raise the feasibility of targeting YAP to interfere with hypoxia-related malignancy including sorafenib resistance. According to our previous study, Statins, the inhibitors of hydroxymethylglutaryl-CoA reductase (HMGCR), could ameliorate hypoxia-provoked nuclear YAP and improve the anti-cancer activity of sorafenib both in vitro and in vivo [9]. However, the mono-treatment of Statins failed to exert robust anticancer activities, which may hamper the clinical application of HMGCR inhibitors in HCC treatment [35]. Therefore, the possibility to combine more potent YAP inhibitor with sorafenib to combat with the hypoxia-caused resistance deserve to be further explored.

CT-707 is a novel multikinase inhibitor that was recently approved by the NMPA for clinical trial in NSCLC. Preclinical study found that CT-707 shows anti-cancer activities against different cancer models, including inhibition of both tumor growth and metastasis. Our previous study showed that CT-707 displayed an ability which inhibits the nuclear translocation of YAP on HCC models [36], so there is a possibility that CT-707 can overcome sorafenib-resistance in hypoxia by inhibiting the dephosphorylation and nuclear translocation of YAP, and the present study demonstrated the hypoxia-mediated sorafenib resistance indeed abated by the co-exposure to CT-707, accompanied with the apoptosis induction [13].

## Conclusions

In summary, our findings demonstrated that the combination of sorafenib and CT-707 exerted synergistic in vitro activity in hypoxic HCC cells. Further studies showed that CT-707 inhibited the activation of YAP through interfering with the dephosphorylation and nuclear translocation of YAP under hypoxia, which would reverse the sorafenib-resistance in HCC under hypoxia (Fig. 4B). And our study provides a promising therapeutic strategy for HCC and expands the horizon for the clinical applications of improving the effect of sorafenib in advanced HCC patients.

## Supplementary Information

**Additional file 1:**  **Supplemental figure 1.** Bel-7402 cell wastreated with serial concentrations of sorafenib in normoxia and hypoxia andcell survival were detected using SRB assay. Data are representative of 3independent experiments and are expressed as the mean ± SD. The symbols *** *P* < 0.001. **Supplemental figure 2. **Bel-7402 cell was treated with sorafenib (10 μM), CT-707(3 μM) or both, and thecell density was observed by optical microscope. **Supplemental figure 3.** Bel-7402 cell was treated with sorafenib (15μM), CT-707(4 μM) or both, and the cell apoptosis was detected by AV/PIstaining (the units of the y-axis and x-axis are fluorescence intensity.y-axis: PI staining; x-axis: Annexin V).

**Additional file 2.**

## Data Availability

The data used to support the findings of this study are available from the corresponding author upon reasonable request.

## Abbreviations

HCC: Hepatocellular Carcinoma
PFS: Progression-Free Survival
SRB: The sulforhodamine B (SRB) assay
YAP: Yes Associate-Protein
VEGFR: Vascular Endothelial Growth Factor Receptors
NMPA: National Medical Products Administration
PARP: Poly (ADP-ribose) polymerase
TCA: Trichloroacetic Acid
PI: Propidium Iodide
AV-PI: Annexin V-PI
IC_50_: Inhibitory Concentration
TTP: The Time to Progression
OS: Overall Survival
HIF-1α: Hypoxia-inducible factor 1-alpha
FOXO3: Forkhead Box O3
HMGCR: Hydroxymethylglutaryl-CoA Reductase
NSCLC: Non-Small Cell Lung Cancer
