# Supplementary figures and images for: CT-707 overcomes hypoxia-mediated sorafenib resistance in Hepatocellular carcinoma by inhibiting YAP signaling
Source: BMC Cancer. 2022 Apr 19;22:425. doi: 10.1186/s12885-022-09520-5 (PMC9020089; doi:10.1186/s12885-022-09520-5)

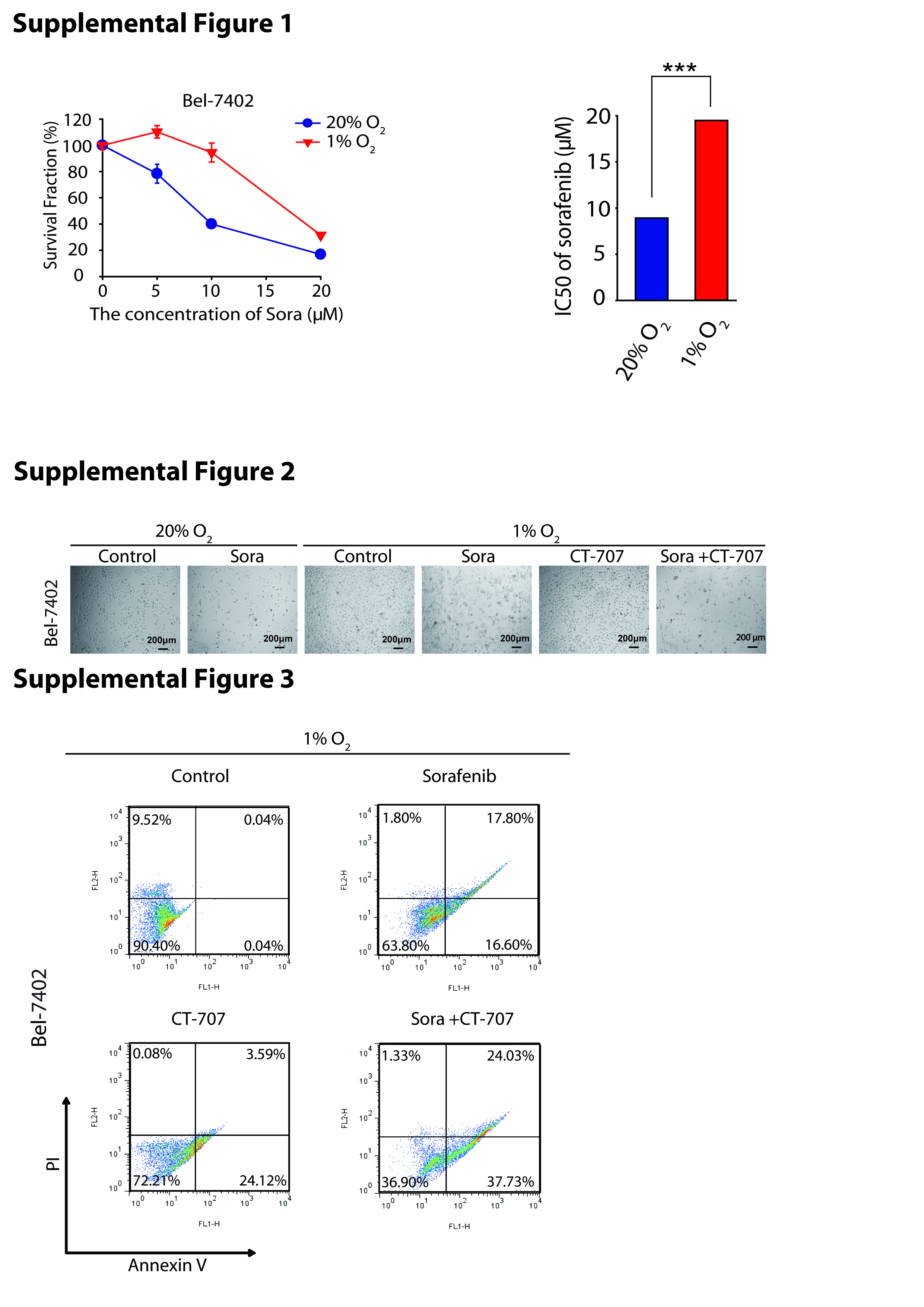

Supplement: Supplementary file 1 — Additional file 1: Supplemental figure 1. Bel-7402 cell wastreated with serial concentrations of sorafenib in normoxia and hypoxia andcell survival were detected using SRB assay. Data are representative of 3independent experiments and are expressed as the mean ± SD. The symbols *** P < 0.001. Supplemental figure 2. Bel-7402 cell was treated with sorafenib (10 μM), CT-707(3 μM) or both, and thecell density was observed by optical microscope. Supplemental figure 3. Bel-7402 cell was treated with sorafenib (15μM), CT-707(4 μM) or both, and the cell apoptosis was detected by AV/PIstaining (the units of the y-axis and x-axis are fluorescence intensity.y-axis: PI staining; x-axis: Annexin V). [file 12885_2022_9520_MOESM1_ESM.tif]
