# Supplementary material for: CT-707 overcomes hypoxia-mediated sorafenib resistance in Hepatocellular carcinoma by inhibiting YAP signaling
Source: BMC Cancer. 2022 Apr 19;22:425. doi: 10.1186/s12885-022-09520-5 (PMC9020089; doi:10.1186/s12885-022-09520-5)
Supplement: Supplementary file 2 — Additional file 2. [file 12885_2022_9520_MOESM2_ESM.pptx]

## Slide 1
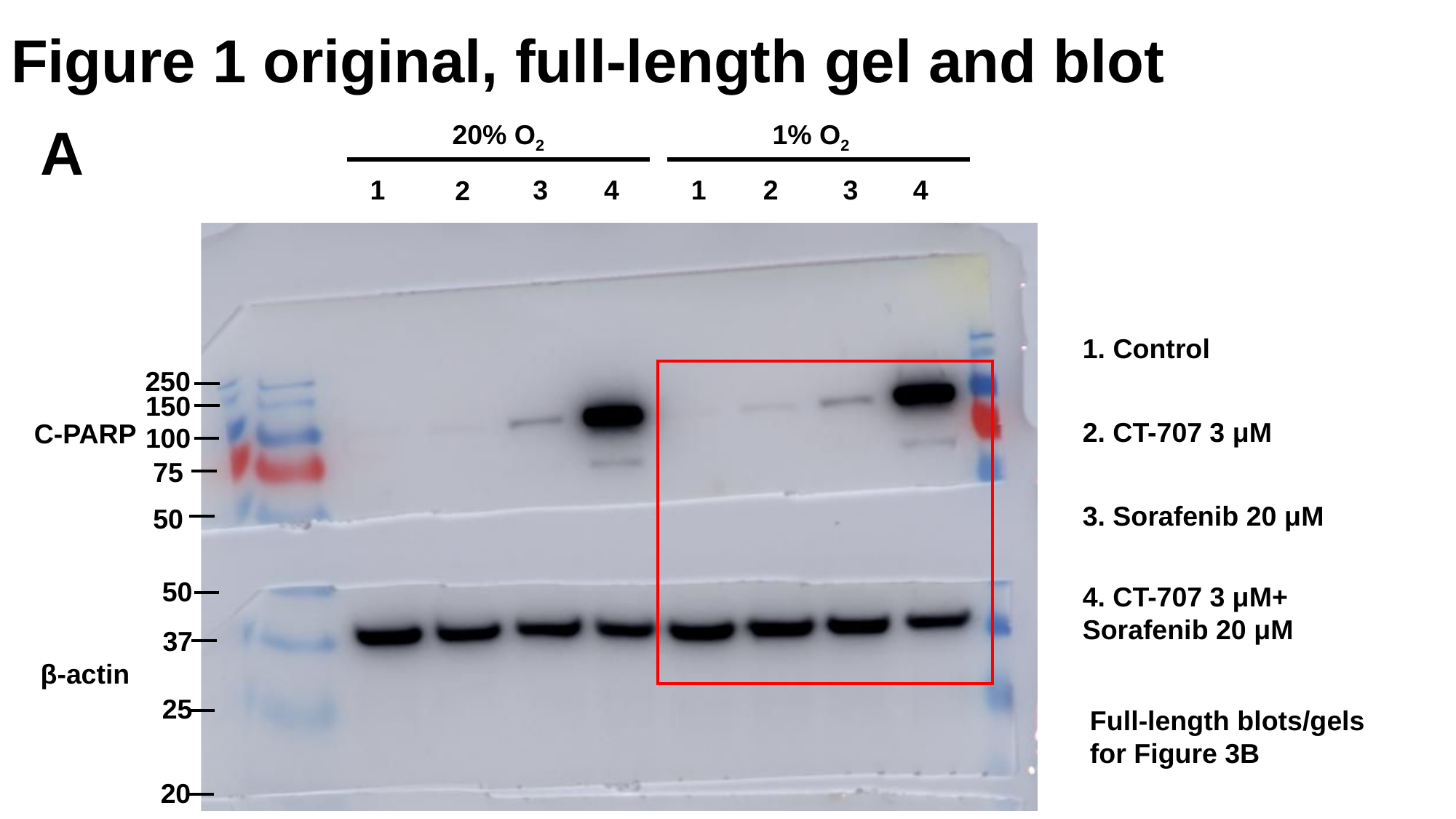

# Figure 1 original, full-length gel and blot
A
20% O2
1% O2
1
3
4
1
2
3
4
2
1. Control
250
150
2. CT-707 3 μM
C-PARP
100
75
3. Sorafenib 20 μM
50
50
4. CT-707 3 μM+ Sorafenib 20 μM
37
β-actin
25
Full-length blots/gels for Figure 3B
20

## Slide 2
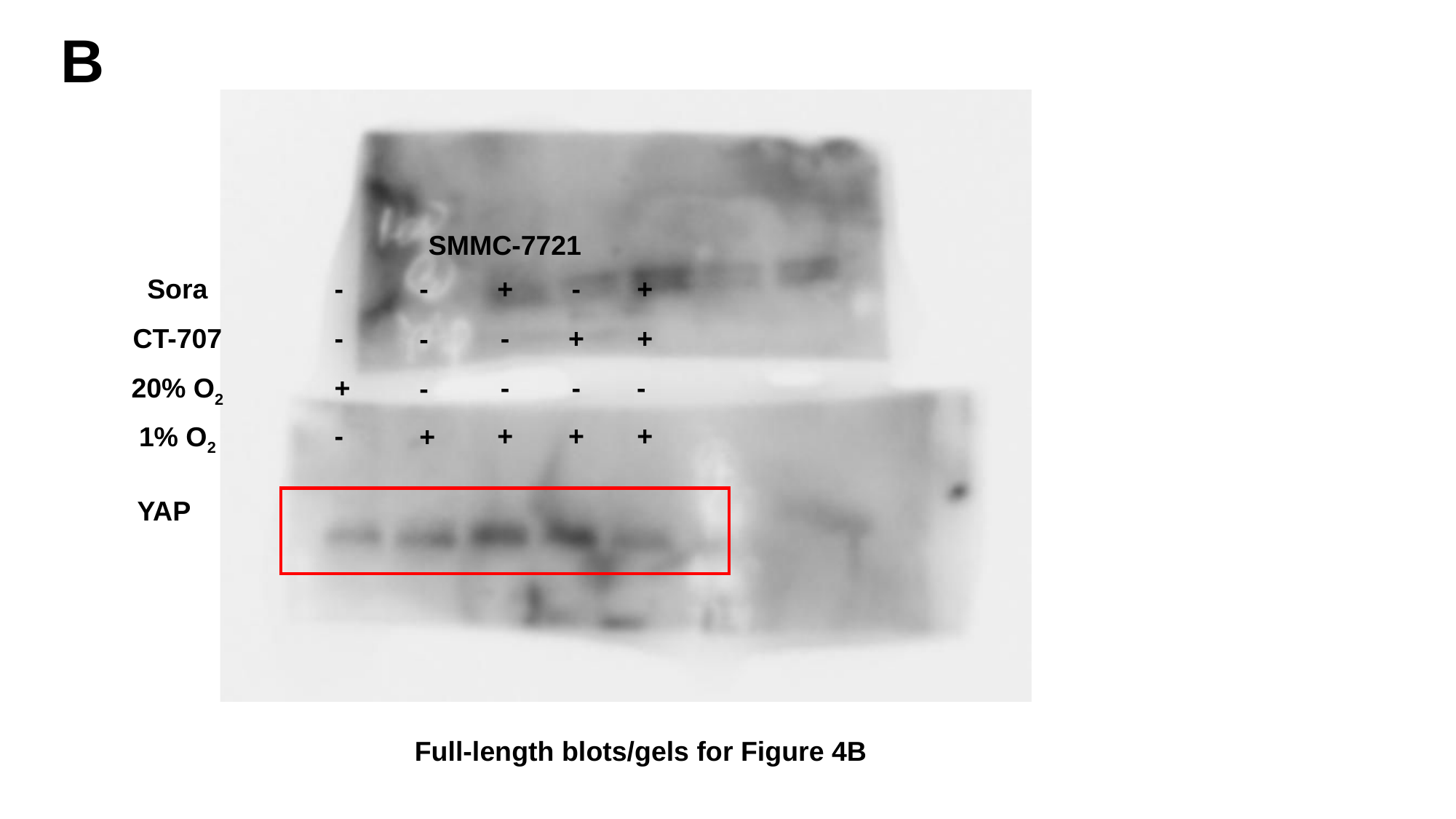

B
SMMC-7721
-
+
-
+
Sora
-
CT-707
-
-
+
+
-
20% O2
+
-
-
-
-
-
+
+
+
1% O2
+
YAP
Full-length blots/gels for Figure 4B

## Slide 3
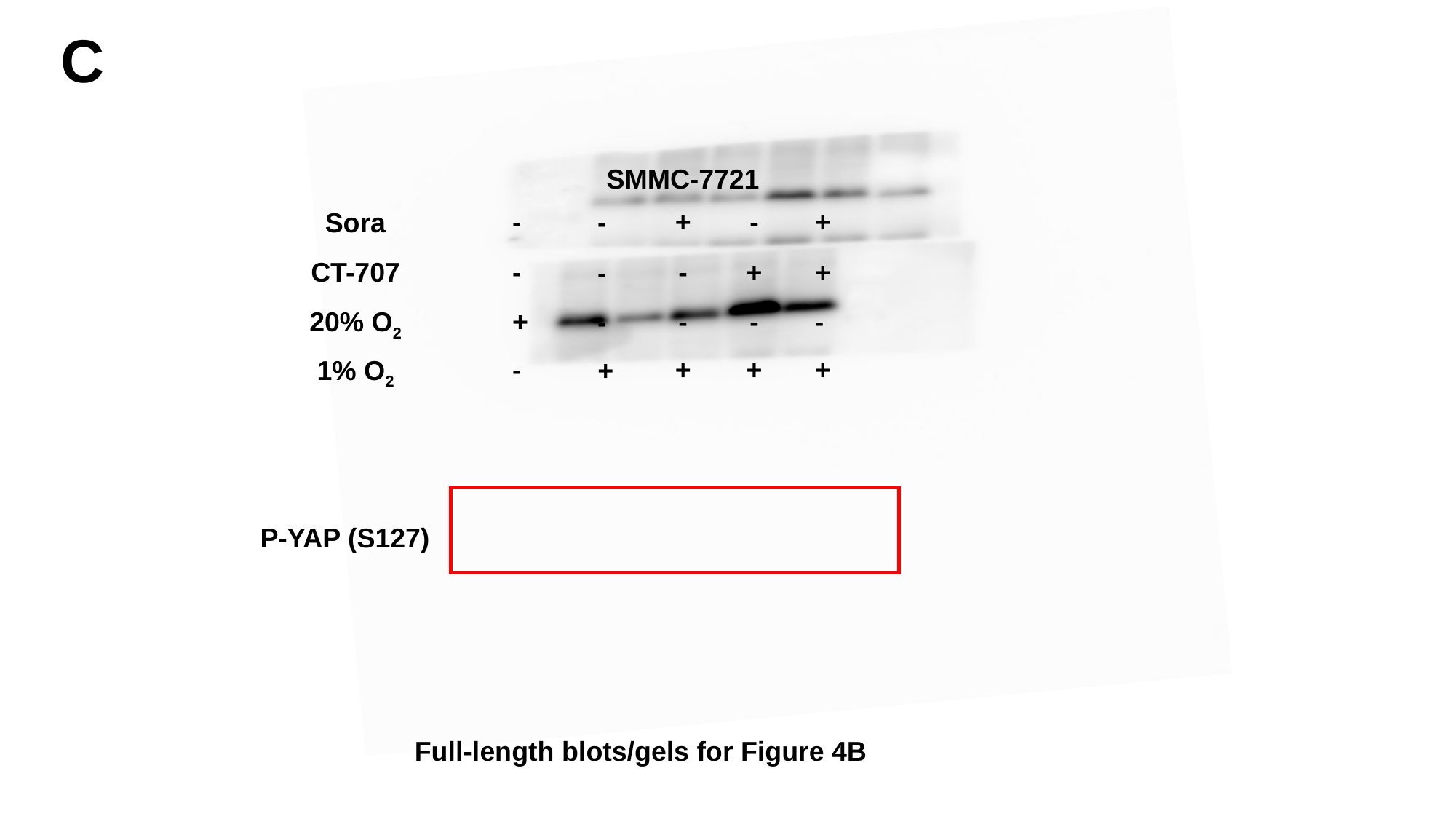

C
SMMC-7721
-
+
-
+
Sora
-
CT-707
-
-
+
+
-
20% O2
+
-
-
-
-
-
+
+
+
1% O2
+
P-YAP (S127)
Full-length blots/gels for Figure 4B

## Slide 4
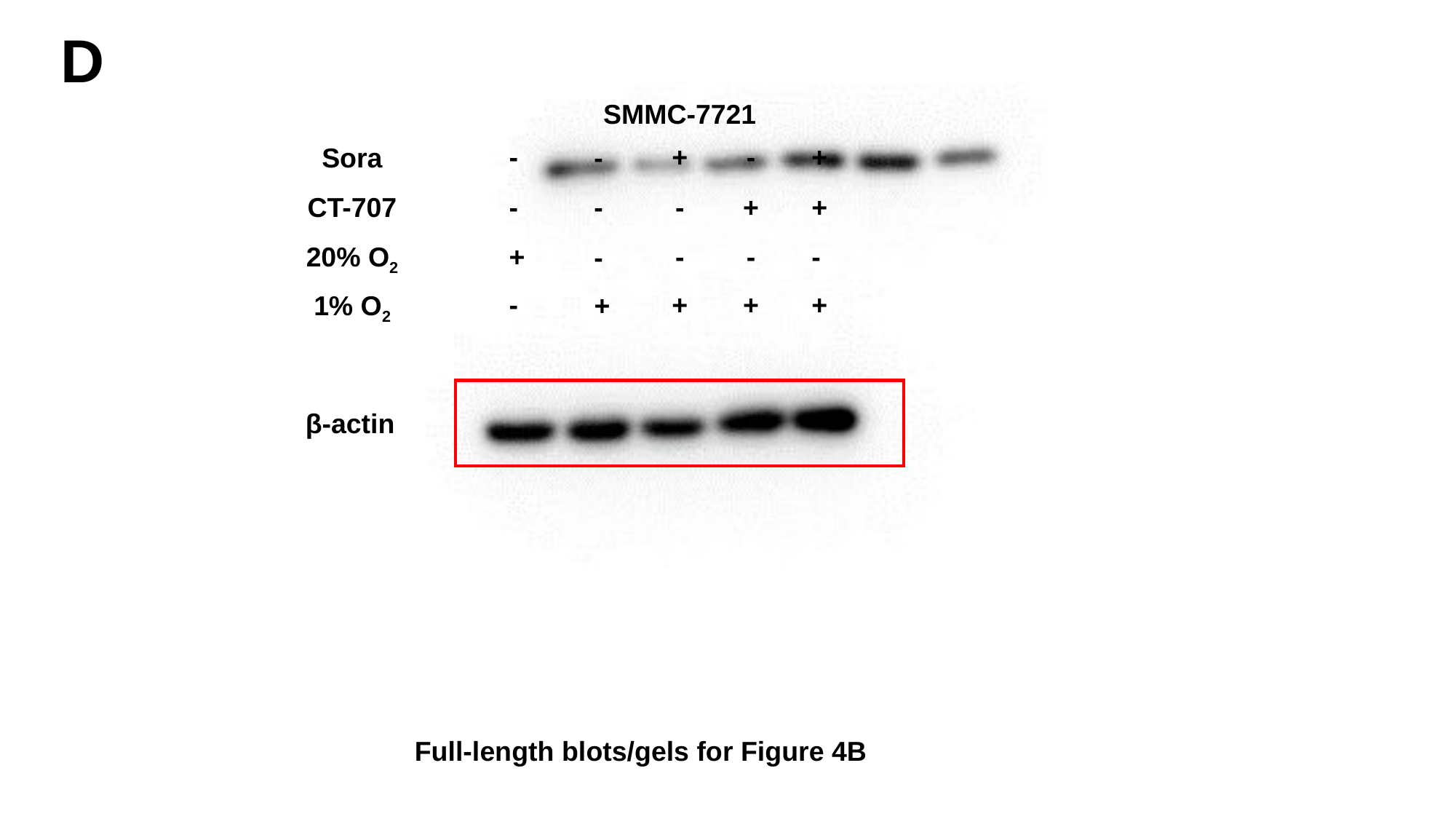

D
SMMC-7721
-
+
-
+
Sora
-
CT-707
-
-
+
+
-
20% O2
+
-
-
-
-
-
+
+
+
1% O2
+
β-actin
Full-length blots/gels for Figure 4B
